# Prognostic Significance of Blood Transfusion in Newly Diagnosed Multiple Myeloma Patients without Autologous Hematopoietic Stem Cell Transplantation

**DOI:** 10.1155/2017/5462087

**Published:** 2017-05-08

**Authors:** Liping Fan, Danhui Fu, Jinping Zhang, Haobo Huang, Qingqing Wang, Yamei Ye, Qianling Xie

**Affiliations:** ^1^Department of Blood Transfusion, Fujian Medical University Union Hospital, Gulou District, Fuzhou City, Fujian Province 350001, China; ^2^Department of Hematology, Fujian Medical University Union Hospital and Fujian Institute of Hematology, Gulou District, Fuzhou City, Fujian Province 350001, China; ^3^Department of Blood Transfusion, Xiamen University Affiliated Fuzhou Second Hospital, Cangshan District, Fuzhou City, Fujian Province 350001, China; ^4^Department of Anesthesia, Macare Women's Hospital in Quanzhou, Quanzhou City, Fujian Province 362000, China

## Abstract

The aim of this study was to evaluate whether blood transfusions affect overall survival (OS) and progression-free survival (PFS) in newly diagnosed multiple myeloma (MM) patients without hematopoietic stem cell transplantation. A total of 181 patients were enrolled and divided into two groups: 68 patients in the transfused group and 113 patients in the nontransfused group. Statistical analyses showed that there were significant differences in ECOG scoring, Ig isotype, platelet (Plt) counts, hemoglobin (Hb) level, serum creatinine (Scr) level, and *β*_2_-microglobulin (*β*_2_-MG) level between the two groups. Univariate analyses showed that higher International Staging System staging, Plt counts < 100 × 10^9^/L, Scr level ≥ 177 *μ*mol/L, serum *β*_2_-MG ≥ 5.5 *μ*mol/L, serum calcium (Ca) ≥ 2.75 mmol/L, and thalidomide use were associated with both OS and PFS in MM patients. Age ≥ 60 was associated with OS and Ig isotype was associated with PFS in MM patients. Moreover, blood transfusion was associated with PFS but not OS in MM patients. Multivariate analyses showed that blood transfusion was not an independent factor for PFS in MM patients. Our preliminary results suggested that newly diagnosed MM patients may benefit from a liberal blood transfusion strategy, since blood transfusion is not an independent impact factor for survival.

## 1. Introduction

Blood transfusion is an important therapeutic tool for patients with critical or malignant diseases. In the last several decades, many studies have shown that blood transfusion improves abnormalities in levels of blood components and increases patients' ability to tolerate therapy, but short-term and long-term adverse effects have also been reported [1]. Several studies showed that components of transfused blood contain several factors that are important for the survival of tumor cells or perturb the recipient's immune system such as VEGF, PDGF-D, tissue plasminogen activator, TGF-*β*, IL-2, IFN-*γ*, and IL-10 [2]. In clinical studies, the effects of blood transfusion on survival in patients with solid tumors remain controversial [3–7].

In hematological malignancies, few studies have examined the relationship between blood transfusion and short-term or long-term adverse effects [8–10]. Jaime-Pérez et al. found that, in children with acute lymphoblastic leukemia (ALL), the number of blood products transfused correlated with poor survival, which may also reflect the severity of the disease [10]. In contrast, Alkayed et al. showed that blood transfusion did not correlate with overall survival (OS) or event-free survival (EFS) in children with ALL [8]. To date, we have not found any studies elucidating the relationship between blood transfusion and survival in patients with multiple myeloma (MM).

In the current study, we reviewed the medical records and follow-up data of newly diagnosed patients with MM in our single center to assess the correlation between blood transfusion on OS and progression-free survival (PFS) of newly diagnosed patients with MM without ASCT.

## 2. Materials and Methods

### 2.1. Ethics Statement

This study was approved by the ethics committee of Fujian Medical University Union Hospital. As this study was retrospective, written informed consent from patients was not sought.

### 2.2. Study Design

A total of 181 newly diagnosed patients with MM without ASCT who had complete follow-up data between June 2010 and June 2015 at our hospital were included in this study.

### 2.3. Acquisition and Definition of Data

In this study, data were collected from the medical records of newly diagnosed patients with MM without ASCT from June 2010 to June 2015 at Fujian Medical University Union Hospital, Fujian, China. Patients who did not receive any therapeutic regimen or received ASCT or those without complete follow-up data were excluded.

Diagnosis and clinical event end points, such as disease progression and relapse, were evaluated by use of the International Myeloma Working Group criteria. OS was measured from the date of diagnosis to the date of death or last follow-up. Death from all causes was included. PFS was measured from the date on which the patient started treatment to the date of disease progression, relapse, or death, whichever came first. Survival time was measured until 31 December 2015.

Patients that received more than 2 units of packed red blood cells (RBC) and/or more than 1 unit (≥2 × 10^11^ platelets per unit) of apheresis platelets (Plt) and/or more than 15 mL/kg of fresh frozen plasma (FFP) during induction, consolidation, and maintenance therapy were categorized as blood transfusion group. All blood products were leukocyte-reduced (leukocyte number < 5 × 10^5^). The storage duration of RBC units ranged from 7 days to 21 days. The storage duration of Plt units and FFP was limited to 5 days and one year, respectively. The decisions to transfuse in patients with MM were made based on the treating doctors' judgment and guided by our hospital's technical manual of clinical blood transfusion [1]. Technical manuals of clinical blood transfusion in our hospital were listed briefly as follows: the RBC transfusion threshold is 60 g/L of hemoglobin, the plasma transfusion threshold is 1.3 times the upper limit of normal or 1.5 times the midpoint of the reference range in standard coagulation screening tests, and the prophylactic Plt transfusion threshold is 10 × 10^9^/L.

### 2.4. Statistical Analyses

Demographic and clinicopathological characteristics were compared between the blood transfusion group and the no-blood transfusion group using the chi-squared test for categorical variables and independent* t*-test for continuous variables. The Kaplan-Meier method was used to calculate survival for PFS and OS, and the log-rank test was used to analyze the significance of differences among these survival curves. Cox regression models were performed for multivariate analyses with adjustments for characteristics that might be significant prognostic factors according to the univariate analyses. All statistical analyses were performed using SPSS 19.0 software. Two-sided *P* values of <0.05 were used as the criterion for statistical significance.

## 3. Results

### 3.1. Characteristics of Transfused and Nontransfused Groups

A total of 181 newly diagnosed inpatients with MM without ASCT were included. The median follow-up interval of all patients was 20.03 (range from 0.3 to 66.73) months. During the follow-up period, 79 deaths occurred.

The demographic and clinicopathological characteristics of all patients before treatment are listed in Table 1. Of the 181 inpatients, 68 patients (37.57%) received a blood transfusion and 113 patients (62.43%) did not. Erythropoiesis-stimulating agents (ESAs) were not used in all 181 patients.

The transfused patients had higher ECOG scores, lower platelet (Plt) counts, lower hemoglobin (Hb) levels, higher serum creatinine (Scr) levels, and higher serum *β*_2_-microglobulin (*β*_2_-MG) levels than nontransfused patients. There was a significant difference in Ig isotype between transfused and nontransfused patients. In the therapeutic regimens that followed their diagnosis, the patients in the transfused group received more bortezomib than nontransfused patients. There were no significant differences in age, gender, ISS staging, serum albumin (Alb) level, serum lactate dehydrogenase (LDH) level, serum calcium (Ca) level, or thalidomide use between the two groups (Table 1).

### 3.2. Univariate and Multivariate Analyses

**Figure 1:**
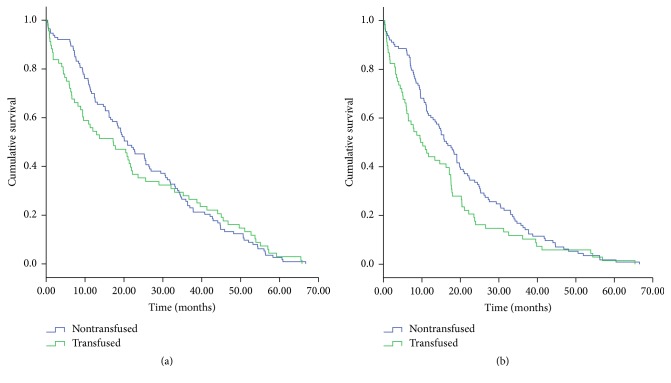
Kaplan-Meier curve of overall survival (a) and progression-free survival (b) of patients with MM who did or did not receive a blood transfusion.

Univariate analyses showed that, in all 181 patients without ASCT, patients with age ≥ 60, higher ISS staging, Plt counts < 100 × 10^9^/L, Scr level ≥ 177 *μ*mol/L, serum *β*_2_-MG ≥ 5.5 *μ*mol/L, or serum Ca ≥ 2.75 mmol/L had shorter OS than others in the cohort. Patients who received thalidomide had longer OS. Blood transfusion was not associated with OS in patients with MM (Table 2, Figure 1(a)).

With respect to PFS in all 181 patients without ASCT, patients with higher ISS staging, Plt counts < 100 × 10^9^/L, Scr level ≥ 177 *μ*mol/L, serum *β*_2_-MG ≥ 5.5 *μ*mol/L, and serum Ca ≥ 2.75 mmol/L and those who received a blood transfusion had shorter PFS. Patients who were treated with thalidomide had longer PFS. Additionally, Ig isotype was associated with PFS (Table 2, Figure 1(b)).

Multivariate analyses showed that age ≥ 60, Plt counts < 100 × 10^9^/L, serum Ca ≥ 2.75 mmol/L, and thalidomide treatment were independent prognostic factors for OS in patients with MM. Moreover, Ig isotype and thalidomide application were independent prognostic factors for PFS in patients with MM. Blood transfusion was not an independent prognostic factor for PFS in patients with MM (Table 3).

## 4. Discussion

MM is an incurable plasma cell disease characterized by the proliferation of malignant monoclonal plasma cells in the bone marrow and accounts for 10% of hematological malignancies. This proportion is expected to increase because of the aging population [11]. Inhibition of hematopoietic function in the bone marrow induced by the proliferation of malignant monoclonal plasma cells and treatment with chemotherapy increased the likelihood of requiring a blood transfusion. Therefore, blood transfusion is an important component of MM therapy.

Several studies have shown that stored blood contains factors that may regulate immune cells, leading to immunosuppression, or promote the survival of tumor cells [2]. However, the effect of blood transfusion on the survival of patients with malignant diseases remains controversial [3–8, 10]. In the solid tumor literature, several studies found that red blood cell transfusion is an independent poor prognostic factor for survival in patients with cancer of the digestive, urinary, or respiratory systems [3, 5, 6, 12–15]. However, others reported that blood transfusion does not affect survival in patients with gastric or renal cell cancer [4, 7]. The effects of transfusion are equally unclear in patients with hematological malignancies [8, 10], such as acute lymphoblastic leukemia as above. Prior to this work, there were no reports on the impact of blood transfusion on survival in patients with MM.

Since ASCT may increase the requirement for blood transfusion, we included 181 newly diagnosed patients with MM who had complete follow-up data and who did not undergo ASCT. Thirty-two newly diagnosed patients with complete follow-up data were excluded because they underwent ASCT. In the current study, we found that, for patients with MM without ASCT, age ≥ 60, higher ISS staging, Plt counts < 100 × 10^9^/L, Scr level ≥ 177 *μ*mol/L, serum *β*_2_-MG ≥ 5.5 *μ*mol/L, serum Ca ≥ 2.75 mmol/L, and the use of thalidomide were correlated with OS, whereas blood transfusion was not associated. In contrast, blood transfusion was associated with PFS in patients with MM without ASCT, as were higher ISS staging, Plt counts < 100 × 10^9^/L, Scr level ≥ 177 *μ*mol/L, serum *β*_2_-MG ≥ 5.5 *μ*mol/L, serum Ca ≥ 2.75 mmol/L, and treatment with thalidomide.

Multivariate analysis showed that blood transfusion is not an independent prognostic factor for PFS in patients with MM without ASCT. After analyzing the characteristics of our cohort, we concluded that, compared with the group of patients who did not require transfusion, higher ISS staging, lower Hb and Plt levels, and renal dysfunction in blood transfusion group led to the requirement for blood transfusion and the lower PFS. Meanwhile, the negative effect of blood transfusion on survival in patients with MM was attenuated by the benefit of bortezomib usage, causing there to be no significant difference in OS between the group that received transfusions and the group that did not. This argument is strengthened by the finding that bortezomib was used at a higher rate in the transfused group than in the nontransfused group, and survival in patients with MM without ASCT is significantly improved by bortezomib [16]. However, lack of data of some important prognostic factors such as fluorescence in situ hybridization (FISH) or cytogenetics in our cohort may influence the results of univariate and multivariate survival analysis. Moreover, hematopoietic failure induced by bortezomib usage may lead to higher transfusion needs, which may influence the analysis of the relationship between blood transfusion and survival in MM patients.

Blood transfusion is an important therapeutic method for patients with MM who are receiving chemotherapy. Hypoxia induced by severe anemia may decrease patients' tolerance to chemotherapy, influence the cytotoxic effect of some drugs such as cyclophosphamide and doxorubicin, and reduce the sensitivity of tumor cells to chemotherapeutics [17]. Meanwhile, protein in plasma may act as a drug carrier and may also influence the metabolism and cytotoxicity of drugs in vivo [18, 19]. Currently, the guidelines regarding thresholds for blood transfusion, including RBC, differ by country. In China, the RBC transfusion threshold is 60 g/L of hemoglobin in nonsurgical patients and 70 g/L of hemoglobin in surgical patients. However, in the United States, a threshold of 70 to 80 g/L of hemoglobin is recommended for patients without underlying cardiac disease, and a threshold of 80 g/L of hemoglobin or less is recommended for patients with underlying cardiac disease [1]. In American and Chinese guidelines, the patient's clinical situation and response to anemia should be taken into account in the decision to transfuse RBC. Similar problems exist with transfusion of other blood products. In our study, though blood transfusion was associated with decreased PFS in patients with MM, it was not an independent negative factor for PFS and OS. Due to the lack of much important data, such as FISH or cytogenetics, relevant to MM patients' prognosis in our cohort, effect of bortezomib on survival and blood transfusion, and the indefinite effects of blood transfusion on survival in patients with tumors [3–7], we think that a prospective study including MM patients who have sufficient data of clinical characteristics and more comparable therapy is needed to evaluate the effects of blood transfusion on MM patients, in order to develop a liberal or restricted transfusion strategy used for decisions about blood transfusion in patients with MM.

In recent years, some studies showed that usage of ESAs could increase the hemoglobin level of MM patients with good tolerance during chemotherapy and might lead to the reduction of RBC transfusion [20, 21]. So, ESAs are recommended as an adjunct to transfusions or alternative in MM therapy to increase the patients' tolerance to chemotherapy and decrease the consumption of blood products.

## Figures and Tables

**Table 1:** Demographic and clinicopathological characteristics of MM patients before treatment.

Characteristic		Transfused (*n* = 68) (%)	Nontransfused (*n* = 113) (%)	*P*
Age	≥60	40 (58.8)	60 (53.1)	0.537
	<60	28 (41.2)	53 (46.9)	
Gender	Male	38 (55.9)	76 (67.3)	0.153
	Female	30 (44.1)	37 (32.7)	
Ig isotype	IgG	40 (58.8)	57 (50.4)	0.208
	IgA	18 (26.5)	29 (25.7)	
	Light chain	10 (14.7)	21 (18.6)	
	Others	0 (0)	6 (5.3)	
ISS staging	I	5 (7.4)	26 (23.0)	0.006
	II	31 (45.6)	55 (48.7)	
	III	32 (47.0)	32 (28.3)	
ECOG	0-1	52 (76.5)	101 (89.4)	0.020
	2–4	16 (23.5)	12 (10.6)	
Plt (×10^9^/L)	≥100	52 (76.5)	104 (92.0)	0.003
	<100	16 (23.5)	9 (8.0)	
Hb (g/L)	≥100	6 (8.8)	49 (43.4)	0.000
	<100	62 (91.2)	64 (56.6)	
Scr (*μ*mol/L)	≥177	24 (35.3)	15 (13.3)	0.001
	<177	44 (64.7)	98 (86.7)	
Serum Alb (g/L)	≥35	13 (19.1)	36 (31.9)	0.062
	<35	55 (80.9)	77 (68.1)	
Serum *β*_2_-MG (*μ*mol/L)	≥5.5	32 (47.0)	32 (28.3)	0.011
	<5.5	36 (53.0)	81 (71.7)	
Serum LDH (IU/L)	≥245	14 (20.6)	14 (12.4)	0.140
	<245	54 (79.4)	99 (87.6)	
Serum Ca (mmol/L)	≥2.75	11 (16.2)	10 (8.8)	0.136
	<2.75	57 (83.8)	103 (91.2)	
Bortezomib	Yes	51 (75.0)	60 (53.1)	0.003
	No	17 (25.0)	53 (46.9)	
Thalidomide	Yes	54 (79.4)	90 (79.6)	0.970
	No	14 (20.6)	23 (20.4)	

Ig: immunoglobulin; ISS: International Staging System; ECOG: Eastern Cooperative Oncology Group; Plt: platelet; Hb: hemoglobin; Scr: serum creatinine; Alb: albumin; *β*_2_-MG: *β*_2_-microglobulin; LDH: lactate dehydrogenase; Ca: calcium.

**Table 2:** Univariate analysis of the correlation between demographic and clinicopathological characteristics and survival time in patients with MM.

Characteristics		*n*	OS (mean)	PFS (mean)
Age	≥60	100	20.355	16.595
	<60	81	28.233	20.102
Gender	Male	114	23.614	18.322
	Female	67	24.333	17.897
Ig isotype	IgG	97	22.531	16.210
	IgA	47	24.419	19.674
	Light chain	31	21.914	18.971
	Others	6	51.645	33.772
ISS staging	I	31	29.096	22.806
	II	86	28.490	21.231
	III	64	15.161	11.796
ECOG	0-1	153	25.098	18.830
	2–4	28	17.230	14.531
Plt (×10^9^/L)	≥100	156	25.251	19.403
	<100	25	15.327	10.440
Hb (g/L)	≥100	55	22.732	21.187
	<100	126	26.511	16.846
Scr (*μ*mol/L)	≥177	39	13.888	10.382
	<177	142	26.625	20.302
Serum Alb (g/L)	≥35	49	25.292	20.049
	<35	132	23.357	17.465
Serum *β*_2_-MG (*μ*mol/L)	≥5.5	64	15.161	11.796
	<5.5	117	28.650	21.648
Serum LDH (IU/L)	≥245	28	18.717	14.700
	<245	153	24.826	18.799
Serum Ca (mmol/L)	≥2.75	21	13.268	11.910
	<2.75	160	25.273	18.986
Bortezomib	Yes	111	24.816	18.957
	No	70	22.398	16.909
Thalidomide	Yes	144	26.712	20.368
	No	37	12.860	9.590
Blood transfusion	Yes	68	22.376	14.998
	No	113	24.786	20.070

**Table 3:** Multivariate analysis of the correlation between demographic and clinicopathological characteristics and survival time in patients with MM.

Covariates	Overall survival		Progression-free survival	
	95% CI for HR	*P*	95% CI for HR	*P*
Age	1.005–1.038	0.011	N/A	N/A
Ig isotype	N/A	N/A	0.659–0.957	0.015
ISS staging	0.697–1.661	0.740	0.694–1.648	0.761
Plt (×10^9^/L)	0.369–0.939	0.026	0.409–1.084	0.102
Scr (*μ*mol/L)	0.977–2.453	0.063	0.975–2.501	0.063
*β*_2_-MG (*μ*mol/L)	0.665–2.524	0.447	0.849–3.244	0.138
Ca (mmol/L)	1.172–3.389	0.011	0.838–2.279	0.205
Thalidomide	0.277–0.612	0.000	0.286–0.641	0.000
Blood transfusion	N/A	N/A	0.735–1.453	0.849

ISS: International Staging System; Scr: serum creatinine; *β*_2_-MG: *β*_2_-microglobulin; Ca: calcium; HR: hazard ratio; CI: confidence interval; N/A: not available.
